# Simulating potential outbreaks of Delta and Omicron variants based on contact-tracing data: A modelling study in Fujian Province, China

**DOI:** 10.1016/j.idm.2023.02.002

**Published:** 2023-02-18

**Authors:** Yichao Guo, Wenjing Ye, Zeyu Zhao, Xiaohao Guo, Wentao Song, Yanhua Su, Benhua Zhao, Jianming Ou, Yanqin Deng, Tianmu Chen

**Affiliations:** a State Key Laboratory of Molecular Vaccinology and Molecular Diagnostics, School of Public Health, Xiamen University, Xiamen City, 361102, Fujian Province, PR China; b Fujian Provincial Center for Disease Control and Prevention, Fujian Province, PR China

**Keywords:** Contact tracing, Vaccine effectiveness, Variant of concern, Mathematical model, COVID-19

## Abstract

Although studies have compared the relative severity of Omicron and Delta variants by assessing the relative risks, there are still gaps in the knowledge of the potential COVID-19 burden these variations may cause. And the contact patterns in Fujian Province, China, have not been described. We identified 8969 transmission pairs in Fujian, China, by analyzing a contact-tracing database that recorded a SARS-CoV-2 outbreak in September 2021. We estimated the waning vaccine effectiveness against Delta variant infection, contact patterns, and epidemiology distributions, then simulated potential outbreaks of Delta and Omicron variants using a multi-group mathematical model. For instance, in the contact setting without stringent lockdowns, we estimated that in a potential Omicron wave, only 4.7% of infections would occur in Fujian Province among individuals aged >60 years. In comparison, 58.75% of the death toll would occur in unvaccinated individuals aged >60 years. Compared with no strict lockdowns, combining school or factory closure alone reduced cumulative deaths of Delta and Omicron by 28.5% and 6.1%, respectively. In conclusion, this study validates the need for continuous mass immunization, especially among elderly aged over 60 years old. And it confirms that the effect of lockdowns alone in reducing infections or deaths is minimal. However, these measurements will still contribute to lowering peak daily incidence and delaying the epidemic, easing the healthcare system's burden.

## 1 Introduction

The coronavirus disease 2019 (COVID-19) pandemic is still spreading worldwide. In China, the Delta variant (B.1.617.2) of the severe acute respiratory syndrome coronavirus 2 (SARS-CoV-2) was first reported on April 22, 2021 (Ye et al., 2021), then became the dominant strain and caused outbreaks with local transmission across China up till December 2021 (Wu et al., 2022; M. Zhang, Xiao, et al., 2021; Zhou et al., 2021). Afterward, the highly contagious Omicron variant was introduced and disseminated throughout China. From March 1 through April 18, 2022, 497,214 local Omicron infections have been spilled over to 31 provinces in mainland China except for Tibet Autonomous Region, generating multiple waves of COVID-19 epidemic (National Health Commission of China, 2022).

China began to provide free COVID-19 vaccine shots in December 2020 (Zheng et al., 2021). The largest share was the inactivated vaccines against SARS-CoV-2 by two manufacturers, Sinovac (CoronaVac) and Sinopharm (BBIBP-Cov). In real-world analyses, it has been observed that a single dose of inactivated vaccine was not sufficiently protective against Delta infection. Two doses of inactivated COVID-19 vaccines were effective against symptomatic when the Delta variant was prevalent (Kang et al., 2022; Wu et al., 2022). Meanwhile, studies have also shown that inactivated vaccines provide sufficient protection against severe COVID-19 illness and death even when Delta and Omicron are circulating (Al Kaabi et al., 2021; Jara et al., 2021; McMenamin et al., 2022; Tanriover et al., 2021). However, concerns have been raised about the possibility that inactivated vaccines may follow the same pattern of declining vaccine-induced immunity as findings describing reductions in the effectiveness of mRNA-based vaccinations against infection as a function of time (Abu-Raddad, Chemaitelly, & Bertollini, 2022; Chemaitelly et al., 2021; Cohn et al., 2022; D.-Y. Lin, Gu, et al., 2022; Tartof et al., 2021). Test-negative, case-control studies in Brazil validated the waning effectiveness of CoronaVac against both Delta and Omicron infection (Cerqueira-Silva et al., 2022; Ranzani et al., 2022).

Although several studies have compared the relative severity of Omicron and Delta variants by assessing the relative risks (Nyberg et al., 2022; Ulloa et al., 2022; Veneti et al., 2022), there are still gaps in the knowledge of the potential COVID-19 burden these variations may cause. And the contact patterns in Fujian Province have not been described. Since changes in social contact patterns shaped the dynamics of the COVID-19 outbreak, they can guide and inform more realistic representations of parameters in mathematical models for infectious diseases. According to studies, lockdowns or social distancing alone are sufficient to contain sporadic COVID-19 during the circulation of wild-type SARS-CoV-2 (Zhang et al., 2020; J. Zhang, Xiao, et al., 2021). More information on whether measures like lockdown alone remain sufficient to control Delta or Omicron transmission and death would help to inform mitigation strategies.

Here, we estimated the effectiveness of inactivated vaccines after full vaccination in the context of a Delta outbreak in Fujian Province, China. We analyzed the contact pairs tracked over the outbreak period and quantified age-mixing contact patterns among different contact settings. Specifically, we developed a multi-group mathematical model to simulate and compare transmission and death of potential Delta and Omicron outbreaks in Fujian Province based on vaccination status and contact patterns extracted from contact tracing data.

## 2 Material and methods

### 2.1 Setting and subjects

In a Delta variant outbreak that began in Putian City and spread to other cities in Fujian, China, from September 4 to September 30, 2021, close contacts were linked to reverse transcriptase-polymerase chain reaction (RT-PCR) tests confirmed COVID-19 index cases. An imported case from abroad sparked this outbreak. However, it was initially detected at a primary school in a sample nucleic acid test conducted by the Putian government after the autumn school opening. All cases were then promptly traced and isolated according to national and provincial protocols for COVID-19 prevention and control (eighth edition) (National Health Commission of the People's Republic of China, 2021*.*). There was no previous infection in all cases. Close contacts were managed to be traced as well and quarantined. Close contacts include those who lived in the same household or stayed in the same public space without protection within close proximity in the two days before symptom onset for symptomatic cases or sampling of the first positive specimen for asymptomatic cases (the detailed identification principles were the same as previous literature mentioned) (Kang et al., 2022). The Fujian Provincial Center for Disease Control and Prevention (Fujian CDC) compiled the contact-tracing database, including index cases and their close contacts. Demographic information and vaccination status were obtained for both. Additionally, the date of symptom onset, date of isolation order issued, clinical outcomes, PCR cycle threshold (Ct) values, date of PCR test, and occupations were obtained for infected individuals. And the age-structured population of Fujian province was sourced from the Statistical Yearbook in 2021 (Statistical Yearbook of Fujian Province, China., 2021).

### 2.2 Vaccination status

The contact-tracing database provided vaccination status (i.e., vaccine type, number of doses received, and corresponding date of vaccination) from the national medical insurance system for each close contact. Of note, no close contacts who were involved in this outbreak received a booster dose. The fully vaccinated group consisted of individuals exposed to infected people after 14 days had elapsed since their second dose. Since the exact date of exposure was quite difficult to identify, we used the time from a close contact receiving the second dose to the exact date of its index case's symptom onset to determine the fully vaccinated group instead. We did a sensitivity analysis to determine the fully vaccinated group, using the time since close contact receiving the second dose to the exact date of its related index case being isolated. The partially vaccinated group consisted of those who received the first dose or people who received the second dose within 14 days before the exposure. The unvaccinated group consisted of individuals who had not received any inactivated vaccines prior to exposure to a confirmed case. Analyses from studies showed that partial vaccination with the inactivated vaccine was ineffective against the Delta variant infection (Kang et al., 2022; Wu et al., 2022). We performed a multivariable logistic regression, and the results confirmed no statistically significant difference against infection between the partially vaccinated and unvaccinated groups (see Appendix). Therefore, we paired unvaccinated and partially vaccinated people together and classified them as unvaccinated.

### 2.3 Contact patterns estimation

We analyzed index case and close contact pairs during the entire outbreak period. The overall contact matrix relies on all connections among the contact-tracing data, representing a setting without stringent lockdowns. School and factory contact settings were categorized by extracting close contacts of occupation-specific index cases (students, teachers, and workers). A contact setting combined school and factory closure was developed by removing close contacts generated by index cases with the occupations of students, teachers, and workers. Contact matrices C mentioned above were constructed with seven age groups (0–9 years, 10–19 years, 20–29 years, 30–39 years, 40–49 years, 50–59 years, and more than 60 years old), with its ij-th entry $c_{ij}$ represents the average number of daily contacts produced by members of age group i with members of age group j. And the total number of people in age group i is $N_{i}$. We quantified the uncertainty of contact matrices using a 1000-times bootstrap with replacement on the original dataset. The 95% confidence intervals (CIs) on the mean were calculated by bootstrap sampling and were weighted by the age-dependent population size of the Fujian province.

### 2.4 Statistical analyses

We described the demographic and clinical outcomes of close contacts and index cases in Fujian province, stratified by vaccination status. The Statistical analyses section of the Appendix gives additional information on this (National Health Commission of China, 2021*.*). We established a retrospective cohort to assess the waning vaccine effectiveness against Delta variant associated infection. The vaccination status was classified as time-dependent. It is a covariate that can change over time with all close contacts entering the cohort as having not yet received any dose. And the terminal event was defined as close contact developing symptoms or the last new case reported. Adjusted hazard ratios (aHRs) with 95% CIs were estimated by comparing rates of Delta variant infection among fully vaccinated with unvaccinated individuals by including all measured covariates in the Cox model. Variables in the model were age group (same as contact matrix) and sex of close contacts, the same age group, sex, and days from symptom onset to the isolation of index cases. VE was calculated as: (1 – aHRs) multiplied by 100%. Due to limitation in sample size, vaccine effectiveness analyses were not stratified by age. Statistical comparisons of vaccine effectiveness by time since vaccination were made using the likelihood ratio test and Wald $\chi^{2}$ tests for contrasts within the Cox model. We also performed a sensitivity analysis regarding another determination of the fully vaccinated group (i.e., determining by time since a close contact receiving the second dose to the exact date of its index case being isolated).

We fitted the observed and truncated serial interval (time interval between symptom onsets in an ‘index case-close contact’ pair) with three distributions (Gamma, lognormal, and Weibull) using maximum likelihood estimation. The truncated data removed negative and zero-valued serial intervals. The goodness of fit was assessed using the Akaike information criterion with correction (AICc). The joint distribution of PCR cycle threshold (Ct) values and days since symptom onset was obtained using a two-dimensional kernel density estimation with a normal kernel stratified by vaccination status. Data analysis was conducted using R (version 4.1.2, R Foundation).

### 2.5 Model development

**Fig. 1:**
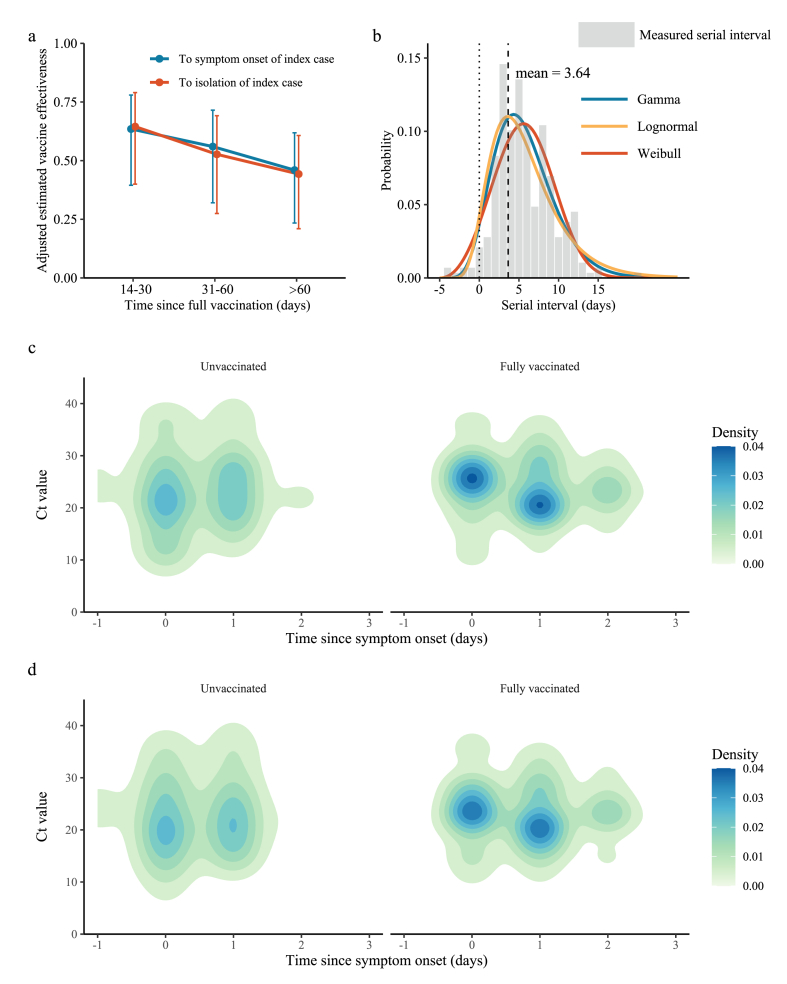
**Duration of vaccine effectiveness and distributions of epidemiology characteristics.** (a) The duration of vaccine effectiveness against Delta variant infection was estimated by adjusted Cox model by time since full vaccination. The blue error bars represent using time since a close contact receiving second dose to the exact date of its index case's symptom onset to determine fully vaccinated group. The red error bars represent using time since a close contact receiving second to the exact date of its index case being isolated to determine fully vaccinated group. (b) Distributions of measured serial interval data with the best fit Weibull distribution based on 286 transmission pairs. (c) Joint distribution of ORF1ab gene PCR cycle threshold (Ct) values and days since symptom onset. (d) Same as (c), but for N gene.

An age and vaccination status specific multi-groups model based on the natural history of COVID-19 was developed. There are six compartments in each group: susceptible (S), exposed (E), pre-symptomatic (P), infectious (I), asymptomatic (A), and recovered/removed (R) (Appendix Fig. 1). The dynamic SEPIAR model with a pre-symptomatic phase (P) was validated by the serial interval distribution fitted in the present study (Fig. 1b, Appendix Fig. 4), since the average serial interval is shorter than the average incubation period, which matches those obtained in earlier studies (Du et al., 2020; Hu et al., 2021; Prete Jr et al., 2021). The pre-symptomatic is a status in transmission dynamics before the symptom onset of the infectious compartment I. The SEPIAR model was based on several assumptions (Model development section in Appendix).

### 2.6 Parameter estimation

Parameters of natural history, including $\kappa_{1},\kappa_{2},\mu ,{1/\omega ,1/\omega }^{\prime },1/\omega^{\prime \prime },1/\gamma$ and ${1/\gamma }^{\prime }$ are defined in Appendix Table 4. According to two systematic reviews, the relative transmission rate from pre-symptomatic and asymptomatic to symptomatic was 0.58 (95% CI, 0.34 to 0.99) and 0.63 (95% CI, 0.18 to 2.26), respectively (Buitrago-Garcia et al., 2020; Byambasuren et al., 2020). Analyses regarding the proportion of asymptomatic SARS-CoV-2 infections have been heterogeneous. A series of systematic reviews and meta-analyses revealed that the proportion of people who tested positive for COVID-19 who never exhibited symptoms ranged from 8.44% (95% CI, 5.12 to 13.62) to 39% (95% CI, 20.4 to 61.4) (Buitrago-Garcia et al., 2020; Byambasuren et al., 2020; Chen et al., 2021; He et al., 2021; Syangtan et al., 2021). The asymptomatic proportion of Delta variant infection (33%) we used in this study was determined from infections in mainland China from April 22, 2021, to August 8, 2021, as displayed in a reference (Gao et al., 2021). And the asymptomatic proportion did not differ between fully vaccinated and unvaccinated groups (Singanayagam et al., 2022). Another study conducted simulations and hypotheses on parameters ${1/\omega ,1/\omega }^{\prime },{1/\omega }^{\prime \prime },1/\gamma$ and ${1/\gamma }^{\prime }$, the days from exposed to asymptomatic and pre-symptomatic (${1/\omega ,1/\omega }^{\prime }$) were both set to 3 (2–5), which was adopted from Delta outbreaks in Guangzhou city and Hunan province by Monte Carlo simulations. The general linear model was also adapted to fit the parameter 1/γ to 5 (4–6), representing days from symptomatic to removed (Chen et al., 2022). Because asymptomatic individuals are more difficult to identify than symptomatic cases, they are expected to wander around before an isolation order is issued for a longer time. Based on this assumption, ${1/\gamma }^{\prime }$ was set to 7 days. The case fatality rate (CFR) caused by Omicron was derived from the epidemic situation announced by the Center for Health Protection of the Department of Health in Hong Kong as of April 6, 2022 (The Centre for Health Protection of the Department of Health, 2022)*.*). The report provided CFR by age group and vaccination status since the fifth wave. According to studies that estimate vaccination against hospital admission and deaths, the effectiveness showed no significant waning for several months after the second dose (Abu-Raddad, Chemaitelly, & Bertollini, 2022; D.-Y. Lin, Gu, et al., 2022; Tartof et al., 2021). It is legitimate to assume that vaccination against death remained stable after full immunization. No deaths occurred in the study population of contact-tracing data we used. The CFR caused by Delta was set proportionally to the Omicron variant, as a study suggested that Omicron had a 69% lower risk of death than Delta cases. Strong evidence of this risk reduction was seen in all age groups (Nyberg et al., 2022). The CFRs are summarized in Appendix Table 5. The vaccine effectiveness by time since the second dose against Omicron infection was collected from another report of Omicron-driven fifth wave in Hong Kong (HKUMed ,2022*.*).

We then derivate the expression for the interactive $R_{ij}$ of SEPIAR model through a definition-based method (Appendix) (Guo et al., 2022). The expression is as follows:

(1) $$\begin{array}{c} R_{ij}=\frac{\beta_{ij}N_{j}}{\mu_{i}\omega_{i}+\left(1-\mu_{i}\right)\omega_{i}^{\prime }}\left(\frac{\kappa_{2}\mu_{i}\omega_{i}}{\gamma_{i}^{\prime }}+\frac{\kappa_{1}\left(1-\mu_{i}\right)\omega_{i}^{\prime }}{\omega_{i}^{\prime \prime }}+\frac{\left(1-\mu_{i}\right)\omega_{i}^{\prime }}{\gamma_{i}}\right) \end{array}$$

Coefficient $\beta_{ij}$ is the transmission rate from group i to group j, which can be formularized as the following equation:

(2) $$\begin{array}{c} \beta_{ij}N_{i}=c_{ji}q\sigma_{j} \end{array}$$

where $N_{i}$ is the total number of people in group i, $c_{ji}$ is the ji-th entry of contact matrix C, q is the probability of contracting SARS-CoV-2 from a single contact, $\sigma_{j}$ is the relative susceptibility of group j. We assumed $\sigma_{j}$ to be 1 in every subgroup, considering the whole population is susceptible to SARS-CoV-2 to the same degree without considering the impact of VE.

The matrix of transmission rate among seven age groups (same groups as the contact matrix) can be derived from the expression of $R_{0}$ along with the leading eigenvalue of contact matrix C (Appendix).

### 2.7 Simulation method

We considered the impact of VE in the model framework as relative risks $1-{VE}_{ij}$, among different protection levels of full vaccination to those unvaccinated. These levels include waning VE at 14–30 days, 31–60 days, and >60 days after the second dose. According to a steady weekly vaccination rate, we assume that throughout the one-year simulation period, the proportion of each vaccination group in the susceptible compartment among the entire population of Fujian Province will remain constant. The simulations for different closure settings in the SEPIAR model were performed by deriving transmission rate coefficient β matrices based on age-structured contact matrices. The simulation for the Delta variant was in the context of real-world vaccination effectiveness estimated in this study. For the Omicron variant, we used accessible VEs in the report from Hong Kong. Both Delta and Omicron simulations started in September 2021 and were under the contact patterns estimated from the contact-tracing data in Fujian, China.

## 3 Results

### 3.1 Contact tracing data analysis

Of 288 PCR-positive close contacts, 286 individuals developed symptoms. We identified 7.34% (21/286) negative serial intervals (symptom onset in close contact precedes their index case) and 8.39% (24/286) zero-valued serial intervals. We found that the best fitting distribution of the measured serial interval by AICc was a Weibull distribution with a mean of 3.64 days and a standard deviation of 3.63 days (Fig. 1b). The mean of the truncated serial interval was 4.56 with a standard deviation of 3.11 and a median of 3.89 days, based on a Gamma distribution (Appendix Fig. 4). We also estimated the joint distribution of Ct values and days since symptom onset. As for unvaccinated individuals' density of ORF1ab gene Ct values at the symptom onset date was found to be the highest with the lowest Ct values. And there is a noticeable density appearing before symptom onset (Fig. 1c). However, the highest density with the lowest Ct values was found one day after symptom onset for fully vaccinated people, and no density appeared before symptom onset. Likewise, this difference appears in N gene Ct values (Fig. 1d).

**Fig. 2:**
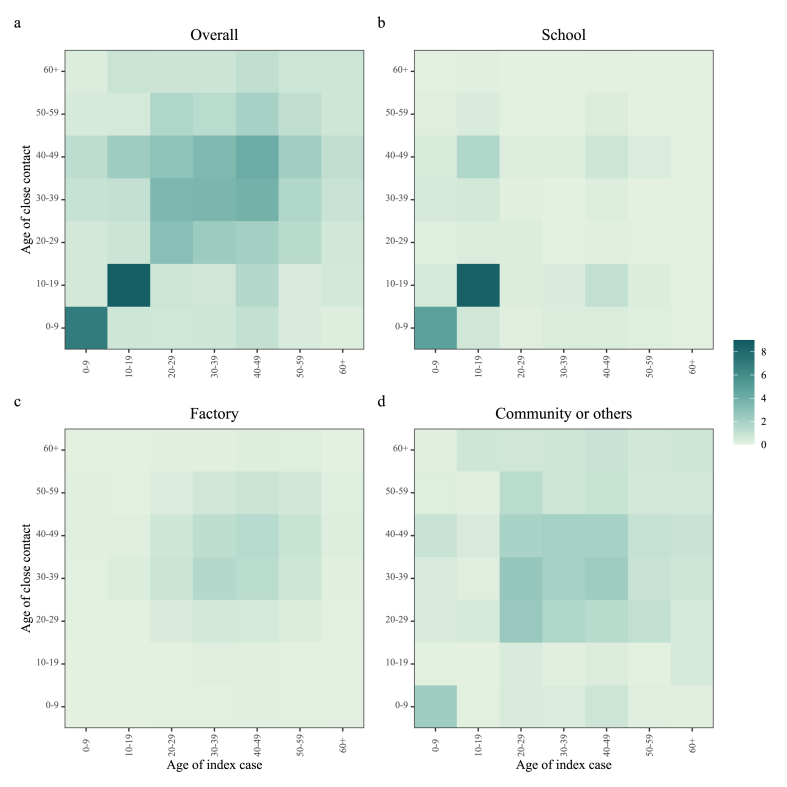
**Contact matrix of index cases and their close contacts.** Each cell of the matrix accounts for the average number of daily contacts produced by a member in each age group with other individuals. The age was categorized to seven groups (0–9 years, 10–19 years, 20–29 years, 30–39 years, 40–49 years, 50–59 years, and more than 60 years old).

We analyzed 8969 close contacts linked to 367 unique index cases based on the two days of contact-tracing for each index case. The age-mixing patterns of overall, school, factory, and community or others contact settings are demonstrated in Fig. 2. The matrix of the overall setting presents an assortative mixing pattern, meaning people tend to meet people of their own age group. It has a mean of 13.26 (95% CI, 12.96 to 13.57) contacts per participant in all age groups, and the highest number of contacts is recorded in the lower left quadrant of the matrix, corresponding to contacts between school-aged children. The contacts of middle-aged people are displayed in the center section of the matrix. The school contact matrix focuses on interactions between students, students and teachers, and teachers, whereas the factory contact matrix depicts interactions between workers. The mean reported contacts in schools is 9.11 (95% CI, 8.78 to 9.44) compared with a mean of 3.59 (95% CI, 3.49 to 3.69) contacts in factories. The last matrix shows the contacts between children aged 0–9 years old as they move around the community, middle-aged people as they interact within household or at other workplaces, and people over 60 years old as they interact within household. In a ‘Community or others’ contact setting, the average number of contacts is 7.57 (95% CI, 7.17 to 7.96).

As shown in Fig. 1a, the effectiveness of inactivated vaccines (Sinovac and Sinopharm) against Delta infection decreased with time since vaccination, declining from 63.49% (95% CI, 39.51%–77.97%, *P* < 0.001) during 14–30 days after receiving the second dose to 56.00% (95% CI, 32.02%–71.52%, *P* < 0.001) during 31–60 days after the second dose. And then, the VE keeps decreasing to 45.96% (95% CI, 23.41%–61.88%, *P* < 0.001) after 60 days. Sensitivity analysis suggests using different time points to determine a fully vaccinated group has only a slight difference in waning VEs (blue and red error bars in Fig. 1a).

### 3.2 Model simulation

**Fig. 3:**
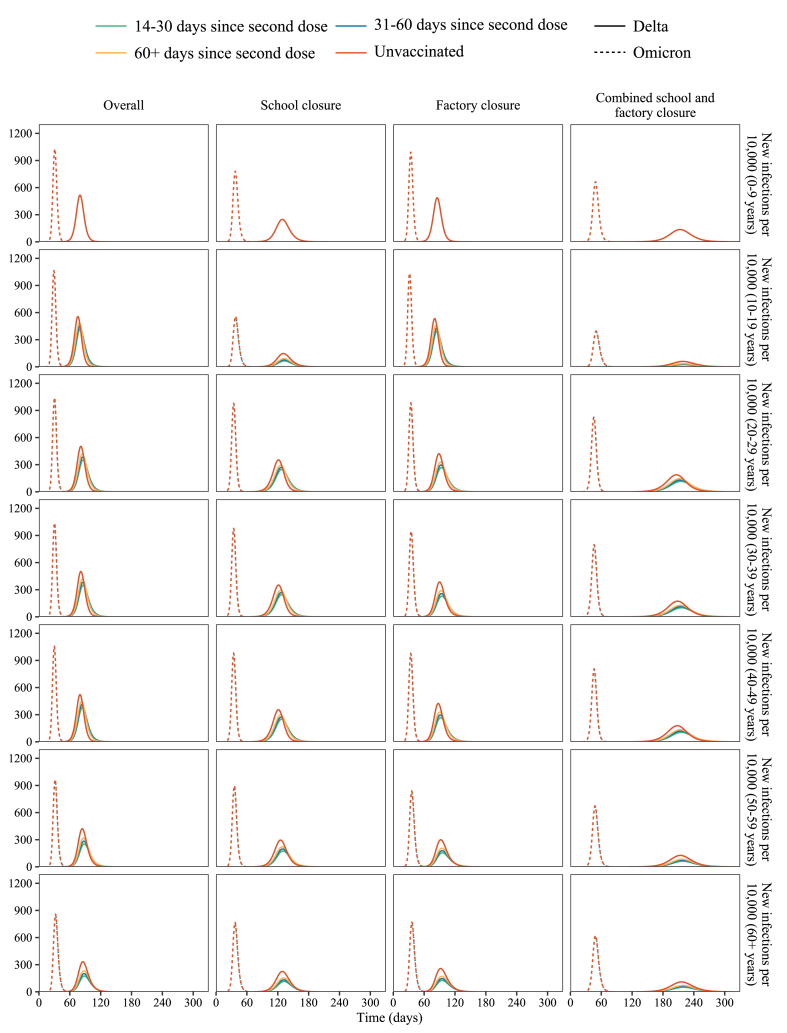
**Simulation for Delta and Omicron variant induced daily new infections per 10,000 (7-day rolling average).** Daily new cases per 10,000 by seven age groups and four vaccination statuses, with solid lines representing VE against Delta infection at 14–30, 31–60, and >60 days after full vaccination corresponding to 63.49%, 56.00% and 45.96%, respectively. Dashed lines represent VE against Omicron infection at 14–30, 31–60, and >60 days after full vaccination corresponding to 0.03%, 0.03%, and 0.01%, respectively.

The projected trend of daily new infections per 10,000 people induced by Delta and Omicron variant are shown in Fig. 3. First, our simulation suggests that adults aged over 60 years in Fujian Province will have the lowest curve of daily new infections, provided there are no restrictions on controlling the contact (i.e., in the ‘Overall’ setting). Second, when compared with the unvaccinated group, all fully vaccinated groups have flattened the incidence curve (reduce peak incidence and delay the epidemic) produced by Delta variant. And with time elapsed since full vaccination, the ‘flattening effect’ is weakened. However, as the dashed lines in Fig. 3 showed, this effect is barely displayed in the Omicron epidemic simulation. Third, in the corresponding setting where contacts in both school and factory are suppressed, the Delta wave ended 358 days after its outbreak. In comparison, the Omicron wave ended 122 days after the outbreak and with roughly twice the number of peak incidences.

**Fig. 4:**
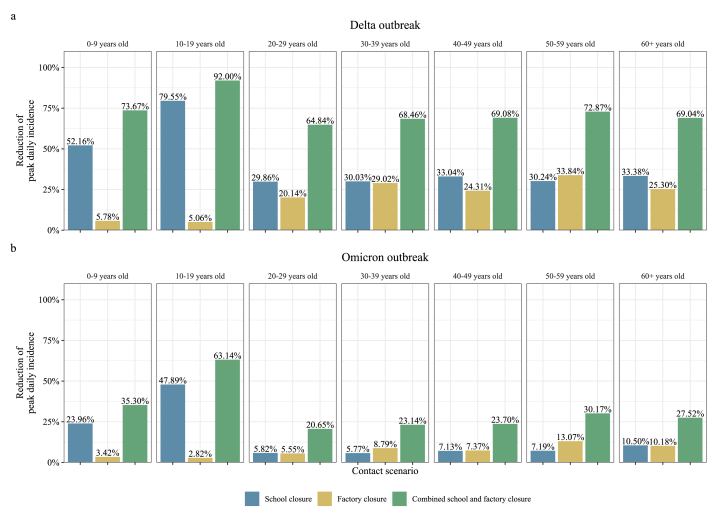
**Reduction of peak daily incidence.** The X-axis represents the proportion of reduction compared with no intervention on reducing contact rates.

As shown in Fig. 4, school closure reduces peak daily incidence the most at 10–19 years old by 79.55% and 47.89% in Delta and Omicron epidemics, respectively. Most reductions of peak daily incidence attributed to factory closure were 33.84% and 13.07% in 50–59 years age group among Delta and Omicron epidemics, respectively. When combining school and factory closure measurements, peak Delta-induced daily incidence reduction ranged from 64.84% to 92.00% across age groups. While during an Omicron wave, this reduction ranged from 20.65% to 63.14%.

**Fig. 5:**
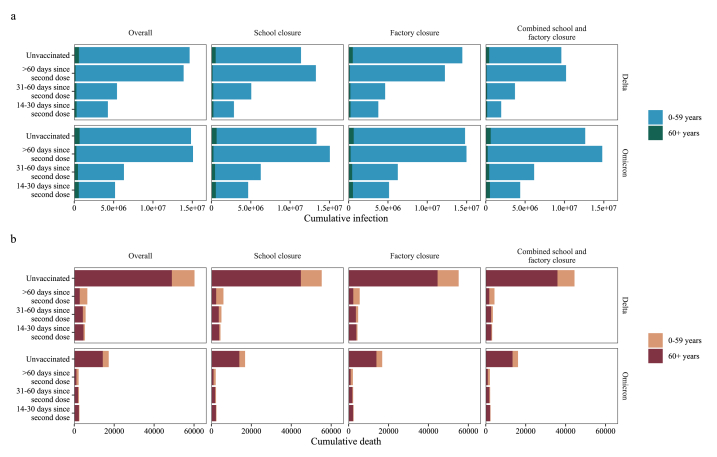
**Cumulative infection and death caused by Delta and Omicron.** Under four contact settings, the cumulative numbers of potential outbreaks were stratified by age group and vaccination status.

We estimated cumulative infection and death caused by Delta and Omicron stratified by contact setting and vaccination status. First, among the four contact settings we constructed, only 3.3%–3.5% of infections would occur in individuals aged >60 years in the Delta wave. It would be between 4.6% and 4.9% in the Omicron wave (Fig. 5a). However, 77.3%–77.8% of the death toll would occur among elderly aged >60 years in the Delta wave. In the Omicron wave, the death toll ranges from 80.3% to 80.5% (Fig. 5b). Second, among the four contact settings, a potential Omicron outbreak corresponds to an increase of 7.6%–32.9% in the number of cumulative infections when compared with Delta variant. In contrast, the cumulative mortality was 0.31–0.41 times the death of Delta variant. Third, compared to the overall contact setting, closing schools and factories simultaneously had limited effects on reducing the death toll of Delta and Omicron by 28.5% and 6.1%, respectively. Nonetheless, the fully vaccinated group exhibited a significant reduction in the death toll. The fully vaccinated group accounted for only 20.0%–22.5% of cumulative deaths given the four contact settings during a Delta wave, and 29.0%–29.2% during an Omicron wave (Fig. 5).

## 4 Discussion

In the present study, we estimated the age-dependent contact matrices using a contact-tracing database in which every close contact is linked to an index case. The average numbers of contacts per day per person were in agreement with questionnaire-based contact surveys conducted in Shanghai in 2018 (Zhang et al., 2019) and Japan in 2014 (Munasinghe et al., 2019). The results were also within the range of contact rates estimated in four Chinese cities (Wuhan, Shanghai, Shenzhen, and Changsha) in 2020 (J. Zhang, Xiao, et al., 2021). Besides, using a contact-tracing database to estimate contact patterns has several advantages compared with the frequently used POLYMOD survey (Mossong et al., 2008). First, the contact-tracing data is less prone to recall bias than the retrospective questionnaire survey (Feehan & Mahmud, 2021; Mossong et al., 2008) due to comprehensive epidemiology investigation and digital information provided by government agencies. Second, the criteria in contact-tracing protocol cover an extensive range of contacts. In contrast, the definition of contact in a questionnaire-based survey was less exhaustive, whether the design was prospective or retrospective (Hoang et al., 2019). However, the contact patterns estimated by the contact-tracing data are limited to the period of the outbreak and may underestimate the contact rate in the absence of lockdowns. In comparison, the POLYMOD survey's contact diary can be readily accepted and is broadly representative of contact patterns on a daily basis (Mossong et al., 2008).

Using a Cox model, our study estimated the waning effectiveness of inactivated COVID-19 vaccines against Delta infections after the second dose. During the Gamma and Delta variant circulation in Brazil, the VE of CoronaVac against infection was estimated using surveillance databases and revealed a temporal trend of 55.0% (95% CI, 54.3 to 55.7) at 14–30 days after the second dose, decreasing to 34.7% (95% CI, 33.1 to 36.2) over 180 days after the second dose (Cerqueira-Silva et al., 2022). Of note, a pattern shared by the VE of mRNA vaccines peaked or remained stable during the first one to two months after full immunization, then fell progressively or rapidly after that (Chemaitelly et al., 2021; Cohn et al., 2022; D.-Y. Lin, Gu, et al., 2022; Tartof et al., 2021). However, the waning VE of inactivated vaccines from the present and the previous study (Cerqueira-Silva et al., 2022) showed only a downtrend.

Among the four contact settings, the Omicron-induced cumulative infection was projected to increase by 7.6%–32.9% compared with the Delta outbreak. In contrast, the cumulative mortality of Omicron was 0.31–0.41 times the death of Delta variant. These results accord with cohort studies which found Omicron infection has a lower risk of severe outcomes than Delta (Hyams et al., 2022; Nyberg et al., 2022). For instance, in the contact setting without stringent lockdowns, we estimated that in a potential Omicron wave, only 4.7% of infections would occur in Fujian Province among individuals aged >60 years, while 58.75% of the death toll would occur in unvaccinated individuals aged >60 years (14,271 people). According to our simulation, the effect of lockdown alone to control infections or deaths caused by Delta or Omicron outbreaks is limited, but the impact of receiving vaccines in reducing the absolute number in death toll was well marked. These findings are comparable to studies that showed that the vaccination campaign provided protection against Delta and Omicron caused mortality (Buchan et al., 2022; L. Lin, Gu, et al., 2022). Additionally, corroborate the findings in larger-scale modelling studies (Bubar et al., 2021; Monod et al., 2021) that the resurgence of COVID-19 was primarily driven by adults 20–49 years of age with highly effective transmission. But the priority of vaccines should be given to the elderly over 60 if reducing fatalities is the goal. Continually stringent lockdowns may not be the best option in the long run, school or factory or even combined school and factory closure cannot sufficiently reduce infection or death on their own. Still, it is important to stress that they can reduce peak incidence and delay the epidemic for months. A study has suggested that implementing such strict intervention can help prevent the collapse of the healthcare system (Cai et al., 2022). Additionally, the joint distribution of Ct values and days since symptom onset suggests that the full vaccination may postpone the peak viral load to after symptom onset. Thus, people who have been fully vaccinated yet have been infected tend to be most contagious when they are at a stage where public health practitioners may easily detect them (due to detectable symptoms) and isolate them in time. An implication of this is that a full vaccination regimen may reduce the proportion of pre-symptomatic transmission, which can significantly help disease control.

In summary, this study validates the need for continuous mass immunization, especially among elderly aged over 60 years old. And it confirms that the effect of lockdowns alone in reducing infections or deaths is minimal. However, these measurements will still contribute to reducing peak daily incidence and delaying the epidemic, easing the healthcare system's burden.

Our study has several limitations. First, hospitalization and death were not used as outcomes in VE evaluation because all COVID-19 patients were admitted to hospital regardless of severity in China in 2021. In the present study, only seven patients became severe illness, and zero death cases, so we referred to the death rate reported in the fifth wave of COVID-19 in Hong Kong, which grouped the population the same as this study applied. Second, none of the close contacts received booster shots before the outbreak in Fujian Province in September 2021. We considered the waning effectiveness by assuming the fraction of each vaccination group in the susceptible compartment is constant over the simulation period. Further studies may consider adding a VE declining function from real-world data partitioned by age groups and vaccination status (with a booster vaccinated group) into mathematical models to guide more realistic representations for SARS-CoV-2 transmission. Third, vaccine coverage varies among provinces due to different speeds of the vaccination campaign, though in general, the problem of lower vaccination coverage among the elderly exists in all regions. Research should be undertaken to refine region-specific vaccination coverages, contact patterns, and migrations between regions, which are instrumental in evaluating region-specific reopening strategies in China.

## Funding

This work was supported by the 10.13039/100000865Bill & Melinda Gates Foundation (INV-005834), 10.13039/501100003392Natural Science Foundation of Fujian Province (NO. 2021J01353 and NO. 2020J01094), and National Science and Technology Major Project of the Ministry of Science and Technology of China (NO.2018ZX10734402-007), Research on accurate prediction and timely response system for outbreaks of new infectious diseases (SRPG2200702) and 10.13039/501100012226Fundamental Research Funds for the Central Universities (No. 20720230001).

## Author contributions

YCG, WJY, ZYZ, and TMC conceived and designed the study. YCG, ZYZ, and XHG drafted the manuscript and interpreted the results. WJY, YQD, and JMO compiled the data. YCG, WJY, ZYZ, WTS, JMO, YQD, and XHG standardized the data and performed the statistical analysis. YCG produced the tables and figures. TMC, JMO, YQD, WTS, YHS, and BHZ provided substantial scientific insight into the interpretation of the results. All authors contributed to revising subsequent versions of the manuscript. All authors read and approved the final manuscript.

## Declaration of interests

All authors declare no competing interests.

## Ethics statement

All data, including demographic, contact-tracing, and immunization information, is collected in accordance with COVID-19 prevention and control measures. This article does not contain any personal information. This study was approved by the institutional ethics committee of the Fujian Center for Disease Control and Prevention, China.

## Code availability

The codes used in this study will be available on GitHub upon manuscript acceptance.

## Declaration of competing interest

The authors declare that they have no known competing financial interests or personal relationships that could have appeared to influence the work reported in this paper.
