# Supplementary material for: Simulating potential outbreaks of Delta and Omicron variants based on contact-tracing data: A modelling study in Fujian Province, China
Source: Infect Dis Model. 2023 Feb 18;8(1):270–81. doi: 10.1016/j.idm.2023.02.002 (PMC9937998; doi:10.1016/j.idm.2023.02.002)
Supplement: Multimedia component 1 [file mmc1.docx]

**Simulating Potential Outbreaks of Delta and Omicron Variants based on Contact-tracing Data: A Modeling Study in Fujian Province, China**

**Appendix**

## Additional Methods

## Vaccination status

| **Appendix Table 1. The result of logistic regression.** | | | | |
| --- | --- | --- | --- | --- |
|  | Unadjusted | | Adjusted* | |
| Vaccination status | RR (95% CI) | VE (95% CI), % | aRR (95% CI) | aVE (95% CI), % |
| Unvaccinated | Reference | ─ | ─ | ─ |
| Partially vaccinated | 0.953 (0.635 to 1.431) | 4.7 (-43.1 to 36.5) | 0.911 (0.556 to 1.495) | 8.9 (-49.5 to 44.4) |
| aRR = adjusted risk ratio; aVE = adjusted vaccine effectiveness; RR = risk ratio; VE = vaccine effectiveness  * Adjusted for sex, age, and occupation. | | | | |

## Statistical analyses

Asymptomatic, mild, moderate, severe, and critically severe were the clinical outcome classifications based on the COVID-19 Diagnosis and Treatment Protocol (eighth edition)^1^. Infections included all classifications from asymptomatic to critically severe. The median time from the second dose to symptom onset of index case was also described for each age group (same as the contact matrix stratified). As for index cases, occupation, days from symptom onset to isolation, and demographic were characterized. We also made statistical comparisons among vaccination statuses, demographic characteristics, and clinical outcomes. The level of statistical significance was defined as *P* < 0.05.

## Model development

**
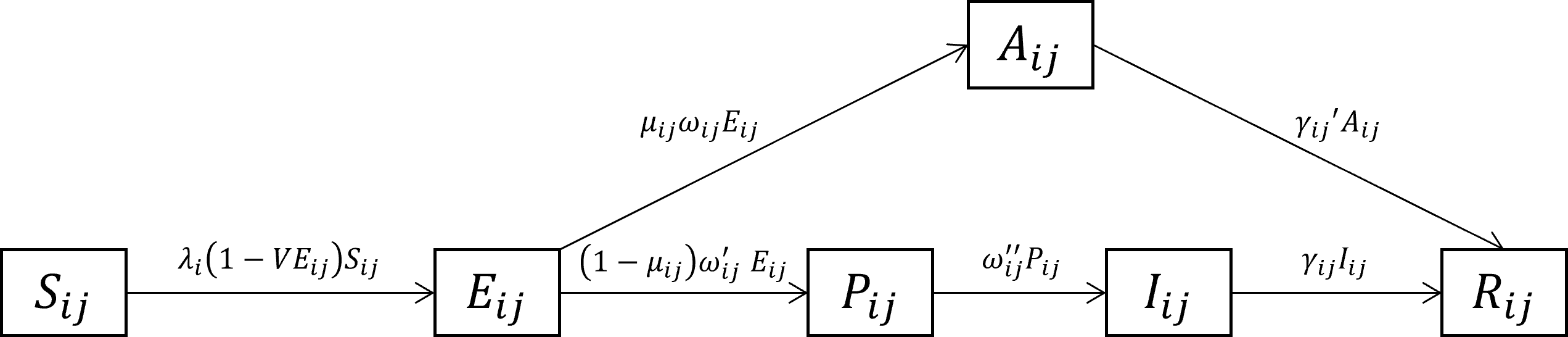
**

**Appendix Figure 1. Flowchart of an age and vaccination status specific multi-group SEPIAR model.** There are six compartments in each group: susceptible (S), exposed (E), pre-symptomatic (P), infectious (I), asymptomatic (A), and recovered/removed (R). Subscripts $i$ and $j$ represent age groups (0 to 9 years, 10 to 19 years, 20 to 29 years, 30 to 39 years, 40 to 49 years, 50 to 59 years, and more than 60 years old) and vaccination status (14-30 days since second dose, 31-60 days since second dose, >60 days since second dose and at most partially vaccinated), respectively.

Assumptions for SEPIAR model:

1. Individuals in the whole society are susceptible to SARS-CoV-2. We denote susceptible individuals as $S_{ij}$, subscript $i$ represents seven age groups: 0 to 9 years, 10 to 19 years, 20 to 29 years, 30 to 39 years, 40 to 49 years, 50 to 59 years, and more than 60 years old, subscript $j$ represents four vaccination statuses: at most partially vaccinated, 14 to 30 days, 31 to 60 days, and >60 days after full vaccination. Thus, the total population in Fujian province is divided into 28 groups.
2. We assume that the proportion of each vaccination status in the susceptible compartment is constant during our one-year simulation period. The proportions, i.e., the initial values of the susceptible are estimated from the contact-tracing data.
3. The relative transmission rate of I, P, and A are set as 1, $\kappa_{1}$ and $\kappa_{2}$, respectively.
4. Transmission of SARS-CoV-2 occurs inside and between each group. We denote the transmission rate coefficient $\beta$ as a matrix. The force of infection $\lambda_{i}$ is defined as:^2^

$$\lambda_{i}=\sum_{k=1}^{7} \beta_{ki}\left[ \sum_{j=1}^{4} \left( I_{kj}+\kappa_{1}P_{kj}+\kappa_{2}A_{kj} \right) \right]$$

5. The latent period from E to A is $1/\omega$, and the incubation period from E to P is $1/\omega^{'}.$ Days from P to I is ${1/\omega}^{''}$. Parameter $\mu$ (0 $\leq\mu\leq1$) represents the proportion of A. Exposed individuals will move out from E into A at a rate of $\mu\omega E$ and P at a rate of $(1-\mu) \omega^{'}E$.
6. Contagious states (P, I, and A) infect susceptible individuals at a rate $\lambda_{i}S_{ij}(1-{VE}_{ij})$ over an infectious period that lasts, on average, for a time $1/{\omega^{''}}, 1/\gamma$ and $1/{\gamma^{'}}$, respectively.

A flowchart of the model is presented in **Appendix Figure 1**. The equations are as follows:

$$\frac{dS_{ij}}{dt}=-\lambda_{i}S_{ij}\left( 1-{VE}_{ij} \right)$$

$$\frac{dE_{ij}}{dt}=\lambda_{i}S_{ij}\left( 1-{VE}_{ij} \right)-\mu_{ij}\omega_{ij}E_{ij}-\left( 1-\mu_{ij} \right){\omega_{ij}}^{'}E_{ij}$$

$$\frac{dP_{ij}}{dt}=\left( 1-\mu_{ij} \right){\omega_{ij}}^{'}E_{ij}-{\omega_{ij}}^{''}P_{ij}$$

$$\frac{dI_{ij}}{dt}={\omega_{ij}}^{''}P_{ij}-\gamma_{ij}I_{ij}$$

$$\frac{dA_{ij}}{dt}=\mu_{ij}\omega_{ij}E_{ij}-{\omega_{ij}}^{'}A_{ij}$$

$$\frac{dR_{ij}}{dt}=\gamma_{ij}I_{ij}-{\gamma_{ij}}^{'}A_{ij}$$

The basic reproduction number ($R_{0}$) is defined as the expectation of secondary cases produced by one infected individual in the entirely susceptible population, during its lifespan as infectious. The definition assumes that in the absence of any intervention,^3^ like quarantine, vaccination, etc. In a multi-group model, the interactive $R_{ij}$ between groups is defined as the expectation of secondary infections that one infected individual in group $i$ will produce in an entirely susceptible population of group $j$ during its lifespan as infectious. All $R_{ij}$ forms a matrix that depicts transmission between groups.


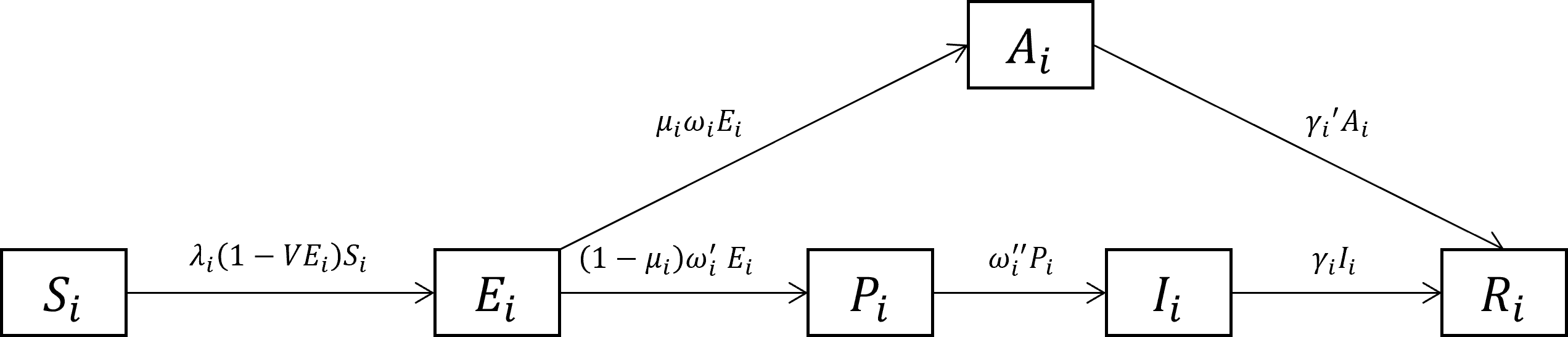


**Appendix Figure 2. Flowchart of an age-specific multi-group SEPIAR model.**

There are six compartments in each group: susceptible (S), exposed (E), pre-symptomatic (P), infectious (I), asymptomatic (A), and recovered/removed (R). Subscript $i$ represents seven age groups: (0 to 9 years, 10 to 19 years, 20 to 29 years, 30 to 39 years, 40 to 49 years, 50 to 59 years, and more than 60 years old).

The steps using a definition-based method^4^ to derivate interactive $R_{ij}$ for SEPIAR model (**Appendix Figure 2**) are as follows:

Step 1: For any infected individual $x_{i}$, there are four possible states: $E_{i}$, ${A_{i}, P}_{i}$ and $I_{i}$. It is shown in the flowchart (**Appendix Figure 2**), that for individual $x_{i}\in E_{i}$, it might develop into $P_{i}$ and $A_{i}$, for individual $x_{i}\in P_{i}$, it will certainly develop into $I_{i}$. Since $\mathbb{P}\left( x_{i}\in E_{i} \right) = 1$, the probabilities are computed by ratio of transition rate given in the flowchart:

$$\begin{matrix} & \mathbb{P}\left( x_{i}\in A_{i} \right)\mathbb{=P}\left( x_{i}\in A_{i}|x_{i}\in E_{i} \right)\mathbb{P}\left( x_{i}\in E_{i} \right)=\frac{\mu_{i}\omega_{i}}{\mu_{i}\omega_{i}+\left( 1-\mu_{i} \right)\omega_{i}^{'}} \\ & \mathbb{P}\left( x_{i}\in P_{i} \right)\mathbb{=P}\left( x_{i}\in P_{i}|x_{i}\in E_{i} \right)\mathbb{P}\left( x_{i}\in E_{i} \right)=\frac{\left( 1-\mu_{i} \right)\omega_{i}^{'}}{\mu_{i}\omega_{i}+\left( 1-\mu_{i} \right)\omega_{i}^{'}} \\ & \mathbb{P}\left( x_{i}\in I_{i} \right)\mathbb{= P}\left( x_{i}\in P_{i} \right)=\frac{\left( 1-\mu_{i} \right)\omega_{i}^{'}}{\mu_{i}\omega_{i}+\left( 1-\mu_{i} \right)\omega_{i}^{'}} \end{matrix}$$

where $\mathbb{P}\left( x_{i}\in E_{i} \right)$ denotes the probability that individual $x_{i}$ will develop into the compartment $E_{i}$; and $\mathbb{P}\left( x_{i}\in A_{i}|x_{i}\in E_{i} \right)$ denotes the conditional probability of $x_{i}$ will develop into the compartment $A_{i}$ with $x_{i}\in E_{i}$ known.

Step 2: Letting one of three components of $\left( {A_{i}, P}_{i}, I_{i} \right)$ equals to 1, and others equal 0 respectively and substitute into the term of newly infection rate $\beta_{ij}S_{j}\left( I_{i}+\kappa_{1}P_{i}+\kappa_{2}A_{i} \right)$, one obtains the number of secondary infections in group $i$, that one infected individual $x_{i}$ will produce per unit time for the different compartments that $x_{i}$ might attain:

$$Q\left( x_{i} \right)=\left\{ \begin{matrix} \beta_{ij}\kappa_{2}S_{j}, & \text{ if }x_{i}\in A_{i} \\ \beta_{ij}\kappa_{1}S_{j}, & \text{ if }x_{i}\in P_{i} \\ \beta_{ij}S_{j}, & \text{ if }x_{i}\in I_{i} \end{matrix} \right.$$

Step 3: Take compartment $I_{i}$ as an example, each infectious individual infects a susceptible individual over an infectious period that lasts, on average, for a time$1/\gamma_{i}$. Similar procedures obtain the infectious period for other compartments of $x_{i}$ might attain:

$$T\left( x_{i} \right)=\left\{ \begin{matrix} 1/\gamma_{i}^{'}, & \text{ if }x_{i}\in A_{i} \\ 1/\omega_{i}^{''}, & \text{ if }x_{i}\in P_{i} \\ 1/\gamma_{i}, & \text{ if }x_{i}\in I_{i} \end{matrix} \right.$$

Step 4: At the beginning of disease transmission, $S\approx N$, and $S/N$ is nearly a constant. As a good 1-order approximation, it is natural to assume that $Q\left( x_{i} \right)$ is constant during the time interval $T\left( x_{i} \right)$.

Step 5: By taking expectations, the expectation of secondary infections in group $j$ that one infected individual in group $i$ will produce during its lifespan as infectious is given by:

$$\begin{matrix} R_{ij}= & \mathbb{P}\left( x_{i}\in A_{i} \right)\times\beta_{ij}\kappa_{2}S_{j}\times1/\gamma_{i}^{'} \\ & \mathbb{+P}\left( x_{i}\in P_{i} \right)\times\beta_{ij}\kappa_{1}S_{j}\times1/\omega_{i}^{''} \\ & \mathbb{+P}\left( x_{i}\in I_{i} \right)\times\beta_{ij}S_{j}\times1/\gamma_{i} \end{matrix}$$

Step 6: Assuming group $ij$ is entirely susceptible, that by letting $S_{j}=N_{j}$, one obtains the $R_{ij}$ defined in the beginning:

$R_{0}$ of the entire population is expressed as the maximum eigenvalue of matrix of all 7 times 7 interactive $R_{ij}$, which is proportional to the infection probability $q$, i.e., a linear equation with one unknown variable $q$. Using $q$ solved by this equation, the transmission rate coefficients $\beta_{ij}$ are reconstructed via its decomposition $\beta_{ij}N_{i}=c_{ji}q\sigma_{j}$.^2^

## Supplementary Results

## Selection of Study Participants

The study period ranged from the first case that developed symptoms of COVID-19 on September 4, 2021, to the last case reported on September 30, 2021. 11,165 persons were identified as close contacts of Delta variant cases. 2,114 close contacts lacking corresponding index case information, one case patient in close contacts missing clinical outcome, and 81 close contacts vaccinated with vaccines other than inactivated vaccine were excluded from the analysis. Resulting in 8,969 close contacts associated with index cases, of whom 3,211 (35.80%) were at most partially vaccinated, but we categorized them as unvaccinated (logistic regression showed that partially vaccinated was not statistically different against infection compared with unvaccinated group), and 5,758 (64.20%) were fully vaccinated. This outbreak was first detected in a primary school, and later 93 students among close contacts were infected. Close contacts under the age of 10 were also included in the study cohort, as they constituted an essential part of the transmission chain (Appendix Figure 3).


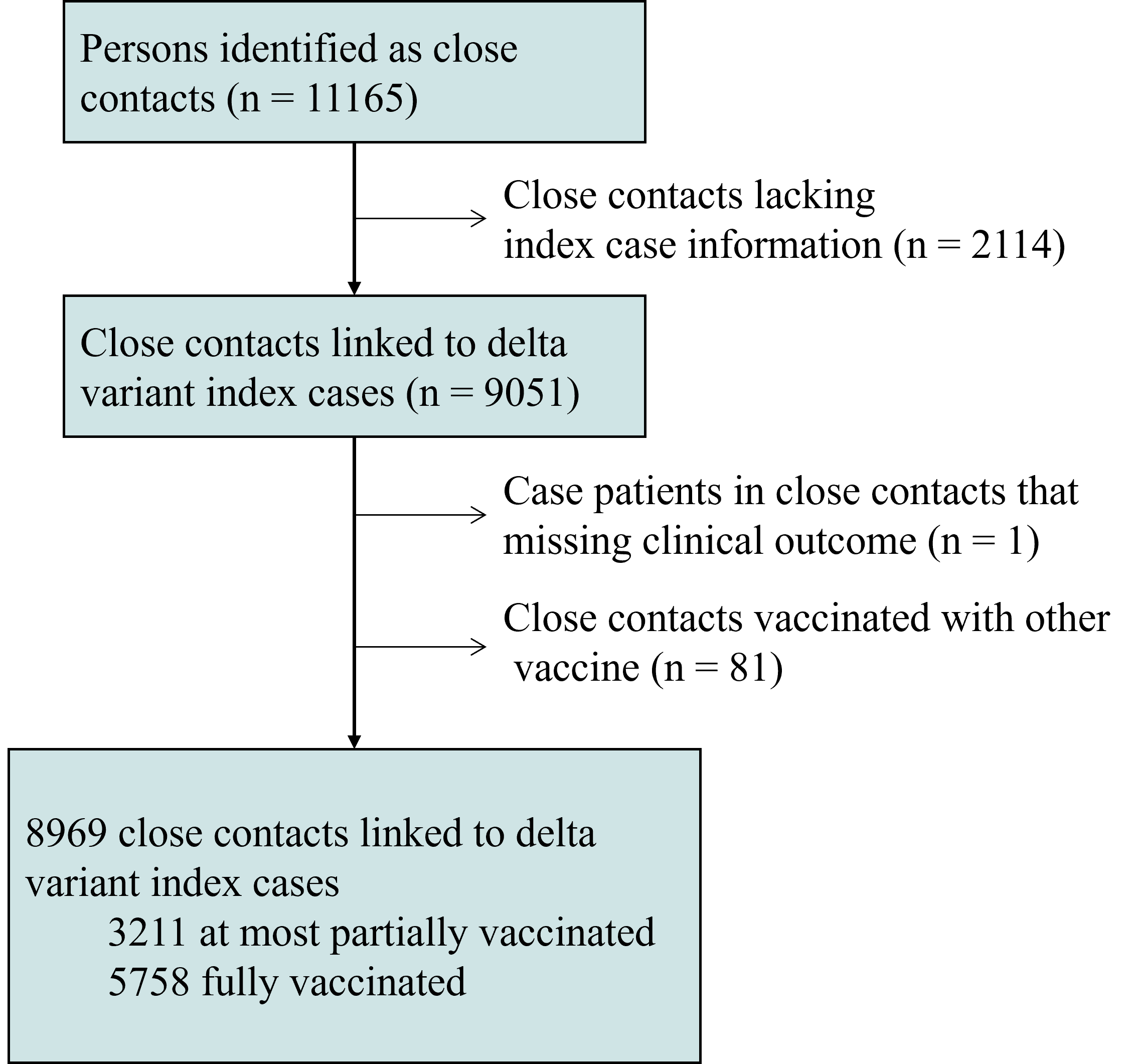


**Appendix Figure 3. Flowchart of selection of study participants identified by Fujian CDC as close contacts of Delta variant cases.** 8,969 close contacts linked to Delta variant index cases were used for vaccine effectiveness estimation, contact matrix construction, and model simulation.

## Epidemiology characteristics

The demographic characteristics, vaccination status, and clinical outcome were summarized in Appendix Table 2. Of 8,969 close contacts, 288 (3.21%) contracted infections (PCR-positive), 3.19% developed symptomatic infections, and 0.08% had severe illness. The median time from PCR-positive patients receive the second dose to the exact symptom onset date of index cases is 80 days (IQR: 44, 88), while among PCR-negative close contacts, this median time is 81 days (IQR: 36, 96). For both PCR positive and negative close contacts, Kruskal-Wallis tests reveal that days from full vaccination to symptom onset of index case differ between age groups (*P* < 0.01). The differences in age and clinical outcome between unvaccinated and fully vaccinated cases are statistically significant (*P* < 0.01, Wilcoxon rank-sum tests). Chi-squared test was used to compare the constituent ratio of sex between two vaccination statuses for both outcomes among close contacts (*P* > 0.05 for PCR-positive contacts, *P* < 0.01 for PCR-negative contacts). The characteristics of 367 unique index cases are demonstrated in Appendix Table 3. Students, teachers, and workers account for more than two-thirds of all index cases. Of these index cases that gave rise to secondary transmission, the proportion who were isolated at least one day after symptom onset made up 53.4%.

| **Appendix Table 2. Characteristics of close contacts.** | | | | | | | |  |
| --- | --- | --- | --- | --- | --- | --- | --- | --- |
| Close contact | Characteristics | Unvaccinated  (n = 3211) | Fully vaccinated (n = 5758) | | | Overall (n = 8969) | p-value | |
| PCR positive | Median time from full vaccination to symptom onset of index case by age, days (IQR) | – | 80 (44, 88) | | – | | <0.01 | |
|  | 0-9 years | – | NA | | – | |  | |
|  | 10-19 years | – | 17 (15.5, 20.5) | | – | |  | |
|  | 20-29 years | – | 82 (45, 93) | | – | |  | |
|  | 30-39 years | – | 82.5 (72, 89.75) | | – | |  | |
|  | 40-49 years | – | 81 (51, 88) | | – | |  | |
|  | 50-59 years | – | 77 (42.5, 84) | | – | |  | |
|  | 60+ years | – | 29.5 (22.75, 37) | | – | |  | |
|  | Median age, years (IQR) | 10 (8.5, 35) | 41 (34, 48) | | 36 (11.75, 46) | | <0.001 | |
|  | Sex |  | |  | |  | 0.2515 | |
|  | Male | 92 (74.8%) | 112 (67.9%) | | | 204 (70.8%) |  | |
|  | Female | 31 (25.2%) | 53 (32.1%) | | | 84 (29.2%) |  | |
|  | Clinical outcome |  |  | | |  | <0.01 | |
|  | Asymptomatic | 2 (1.6%) | 0 (0.0%) | | | 2 (0.7%) |  | |
|  | Mild | 51 (41.5%) | 40 (24.2%) | | | 91 (31.6%) |  | |
|  | Moderate | 66 (53.7%) | 122 (73.9%) | | | 188 (65.3%) |  | |
|  | Severe | 4 (3.3%) | 3 (1.8%) | | | 7 (2.4%) |  | |
|  | Total | 123 | 165 | | | 288 |  | |
| PCR negative | Median time from full vaccination to symptom onset of index case by age, days (IQR) | – | | 81 (36, 96) | | – | <0.001 | |
|  | 0-9 years | – | | NA | | – |  | |
|  | 10-19 years | – | | 25 (20, 28) | | – |  | |
|  | 20-29 years | – | | 81 (40, 97) | | – |  | |
|  | 30-39 years | – | | 86 (43, 99) | | – |  | |
|  | 40-49 years | – | | 88 (47, 116) | | – |  | |
|  | 50-59 years | – | | 85 (43, 96) | | – |  | |
|  | 60+ years | – | | 34 (23.25, 54) | | – |  | |
|  | Median age, years (IQR) | 10 (7, 25) | | 37 (30, 47.75) | | 32 (13, 44) | <0.001 | |
|  | Sex |  | |  | |  | <0.001 | |
|  | Male | 2294 (74.3%) | | 3943 (70.5%) | | 6237 (71.8%) |  | |
|  | Female | 794 (25.7%) | | 1650 (29.5%) | | 2444 (28.2%) |  | |
|  | Total | 3088 | | 5593 | | 8681 |  | |

| **Appendix Table 3. Characteristics of index cases.** | |  |
| --- | --- | --- |
| Characteristics | Unique index case (n = 367) | Percentage |
| Median age, years (IQR) | 35 (16, 44) | ─ |
| Sex |  |  |
| Male | 159 | 43.3% |
| Female | 208 | 56.7% |
| Occupation |  |  |
| Students and teachers | 87 | 23.7% |
| Workers | 162 | 44.1% |
| Others | 118 | 32.2% |
| Time from symptom onset to isolation, days |  |  |
| <0 | 118 | 32.2% |
| 0 | 53 | 14.4% |
| >0 | 196 | 53.4% |

## Results of Serial Interval Estimation


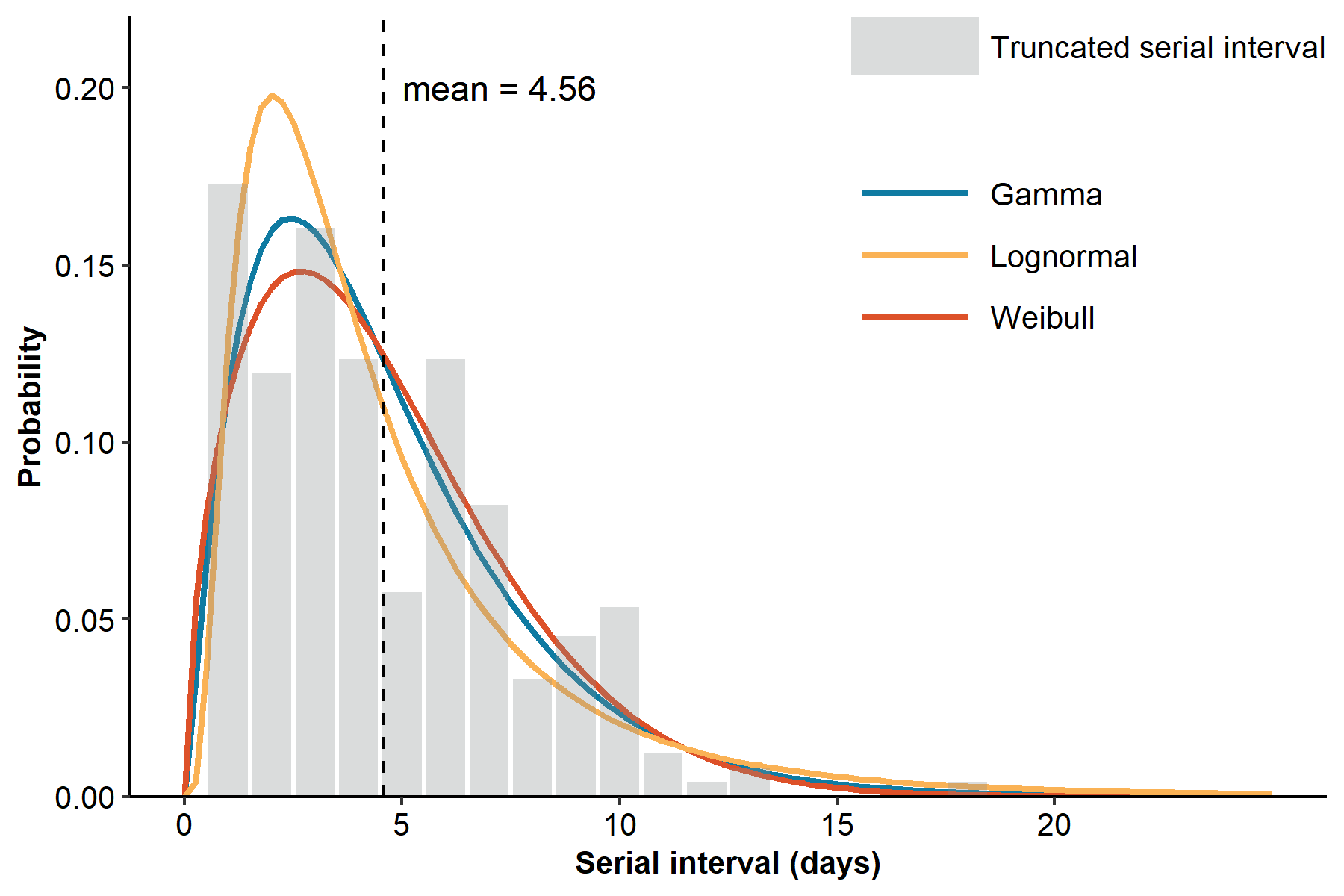


**Appendix Figure 4. Distributions of truncated serial interval.**

Serial interval data truncated to include only positive values with best fit Gamma distribution based on 243 transmission pairs.

| **Parameter estimation**  **Appendix Table 4. Source and value of parameters in the SEPIAR model** | | | | | |
| --- | --- | --- | --- | --- | --- |
| Parameter | Description | Value | | Source | |
|  |  | Delta | Omicron | Delta | Omicron |
| $\kappa_{1}$ | Relative transmission rate from pre-symptomatic to symptomatic | 0.63 | 0.63 | Reference^5^ | Assumption |
| $\kappa_{2}$ | Relative transmission rate from asymptomatic to symptomatic | 0.35 | 0.35 | Reference^5^ | Assumption |
| $\mu$ | Asymptomatic proportion | 0.33 | 0.55 | Reference^5, 6^ | Reference^7-9^ |
| $1/\omega$ | Days from exposed to asymptomatic | 3 | 3 | Reference^10^ | Reference^11, 12^ |
| $1/\omega^{'}$ | Days from exposed to pre-symptomatic | 3 | 2 | Reference^10^ | Reference^11, 12^ |
| ${1/\omega}^{''}$ | Days from pre-symptomatic to symptomatic | 2 | 1 | Reference^10^ | Reference^11, 12^ |
| $f$ | Case fatality rate | - | - | Assumption | Report |
| $1/\gamma$ | Days from symptomatic to removed | 5 | 3 | Reference^10^ | Reference^12, 13^ |
| ${1/\gamma}^{'}$ | Days from asymptomatic to removed | 7 | 3 | Assumption | Reference^12, 13^ |
| $R_{0}$ | The basic reproduction number | 5.08 | 8 | Reference^14^ | Assumption^15^ |

Note: Case fatality rate $f$ is displayed in Table S2.

| **Appendix Table 5. Case fatality rate of Delta and Omicron variant** **by age groups and vaccination status.** | | | | | | | | |
| --- | --- | --- | --- | --- | --- | --- | --- | --- |
| Groups | At most partially vaccinated | | 14-30 days after second dose | | 31-60 days after second dose | | >60 days after second dose | |
|  | Delta | Omicron | Delta | Omicron | Delta | Omicron | Delta | Omicron |
| 0-9 | 0.000322581 | 0.0001 | 6.45161E-05 | 0.00002 | 6.45161E-05 | 0.00002 | 6.45161E-05 | 0.00002 |
| 10-19 | 0.000322581 | 0.0001 | 6.45161E-05 | 0.00002 | 6.45161E-05 | 0.00002 | 6.45161E-05 | 0.00002 |
| 20-29 | 0.000967742 | 0.0003 | 0.000193548 | 0.00006 | 0.000193548 | 0.00006 | 0.000193548 | 0.00006 |
| 30-39 | 0.001290323 | 0.0004 | 0.000258065 | 0.00008 | 0.000258065 | 0.00008 | 0.000258065 | 0.00008 |
| 40-49 | 0.004193548 | 0.0013 | 0.000322581 | 0.0001 | 0.000322581 | 0.0001 | 0.000322581 | 0.0001 |
| 50-59 | 0.016129032 | 0.005 | 0.001290323 | 0.0004 | 0.001290323 | 0.0004 | 0.001290323 | 0.0004 |
| 60+ | 0.125439677 | 0.0388863 | 0.023909113 | 0.007411825 | 0.023909113 | 0.007411825 | 0.023909113 | 0.007411825 |

Note: The case fatality rate (CFR) caused by Omicron was derived from the epidemic situation announced by the Center for Health Protection

of the Department of Health in Hong Kong as of April 6, 2022. According to studies that estimate vaccination against hospital admission and deaths,

the effectiveness showed no significant waning for several months after the second dose.^16-18^ It is legit to assume that vaccination against death

remained stable after full immunization. Since no deaths occurred in the study population of contact-tracing data we used, the CFR caused by Delta

was set proportional to Omicron variant, as a study suggested that Omicron had a 69% lower risk of death than that of Delta cases, and strong

evidence of this risk reduction was seen in all age groups.^19^

# References

1. National Health Commission of China, National Traditional Chinese Medicine Administration. Diagnosis and treatment protocol for COVID-19 patients. In Chinese. (revised tentative 8th edition). 14 April 2021. <http://www.gov.cn/zhengce/zhengceku/2021-04/15/5599795/files/e9ce837932e6434db998bdbbc5d36d32.pdf> (accessed 5 February 2022)

2. Guo X, Liu Z, Yang S, Zhao Z, Guo Y, Abudurusuli G, et al. Optimal vaccination with time-varying based on immunity barrier in Hunan Province, China. medRxiv. 2022.

3. Becker NG, Glass K, Barnes B, Caley P, Philp D, McCaw J, et al. Using mathematical models to assess responses to an outbreak of an emerged viral respiratory disease. Final Report to the Australian Government Department of Health and Ageing National Centre for Epidemiology and Population Health, Australian National University. 2006.

4. Guo X, Guo Y, Zhao Z, Yang S, Su Y, Zhao B, et al. Computing R0 of dynamic models by a definition-based method. Infectious Disease Modelling. 2022.

5. Gao W, Lv J, Pang Y, Li L-M. Role of asymptomatic and pre-symptomatic infections in covid-19 pandemic. bmj. 2021;375.

6. Singanayagam A, Hakki S, Dunning J, Madon KJ, Crone MA, Koycheva A, et al. Community transmission and viral load kinetics of the SARS-CoV-2 delta (B. 1.617. 2) variant in vaccinated and unvaccinated individuals in the UK: a prospective, longitudinal, cohort study. The lancet infectious diseases. 2022;22(2):183-95.

7. Houhamdi L, Gautret P, Hoang VT, Fournier PE, Colson P, Raoult D. Characteristics of the first 1119 SARS‐CoV‐2 Omicron variant cases, in Marseille, France, November− December 2021. Journal of Medical Virology. 2022;94(5):2290-5.

8. Sharma RP, Gautam S, Sharma P, Singh R, Sharma H, Parsoya D, et al. Clinico epidemiological profile of Omicron variant of SARS CoV2 in Rajasthan. medRxiv. 2022.

9. Servellita V, Syed AM, Morris MK, Brazer N, Saldhi P, Garcia-Knight M, et al. Neutralizing immunity in vaccine breakthrough infections from the SARS-CoV-2 Omicron and Delta variants. Cell. 2022.

10. Chen T, Zhao Z, Niu Y, Chu M, Rui J, Wang Y, et al. Feasibility of COVID-19 control from a pandemic to endemic. 2022.

11. COVID C, Team R. SARS-CoV-2 B. 1.1. 529 (Omicron) Variant—United States, December 1–8, 2021. Morbidity and Mortality Weekly Report. 2021;70(50):1731.

12. CDC Updates and Shortens Recommended Isolation and Quarantine Period for General Population. 27 December 2021. <https://www.cdc.gov/media/releases/2021/s1227-isolation-quarantine-guidance.html> (accessed 2 March 2022)

13. Omicron's Incubation Period Is Really Short: Here’s Why That Matters. Jan 24 2022. <https://www.prevention.com/health/a38868608/omicron-incubation-period/> (accessed 2 March 2022)

14. Liu Y, Rocklöv J. The reproductive number of the Delta variant of SARS-CoV-2 is far higher compared to the ancestral SARS-CoV-2 virus. Journal of travel medicine. 2021.

15. Omicron is the Dominant COVID Variant for Two Reasons. 28 Dec 2021. <https://vitals.sutterhealth.org/omicron-is-the-us-dominant-covid-variant-for-two-reasons/> (accessed 2 March 2022)

16. Lin D-Y, Gu Y, Wheeler B, Young H, Holloway S, Sunny S-K, et al. Effectiveness of Covid-19 Vaccines over a 9-Month Period in North Carolina. New England Journal of Medicine. 2022.

17. Tartof SY, Slezak JM, Fischer H, Hong V, Ackerson BK, Ranasinghe ON, et al. Effectiveness of mRNA BNT162b2 COVID-19 vaccine up to 6 months in a large integrated health system in the USA: a retrospective cohort study. The Lancet. 2021;398(10309):1407-16.

18. Abu-Raddad LJ, Chemaitelly H, Bertollini R. Waning mRNA-1273 Vaccine Effectiveness against SARS-CoV-2 Infection in Qatar. New England Journal of Medicine. 2022.

19. Nyberg T, Ferguson NM, Nash SG, Webster HH, Flaxman S, Andrews N, et al. Comparative analysis of the risks of hospitalisation and death associated with SARS-CoV-2 omicron (B. 1.1. 529) and delta (B. 1.617. 2) variants in England: a cohort study. The Lancet. 2022;399(10332):1303-12.
